# A Wrong Righted

**Published:** 1875-07

**Authors:** 


### A WRONG RIGHTED.

We have taken frequent occasion to congratulate our readers upon the rapid growth of the new and vital thought concerning the human feet and their treatment, and have received the earnest thanks of thousands of enfranchised souls for our work in placing before them in our pages the truths which have set them free. We believe that the large majority of our old readers are by this time fully converted to the new and rational system of foot clothing devised by Joel McComber, and now so widely adopted by thoughtful people; and were our influence confined to these solely, we should feel that our whole duty had been accomplished, and that our work was done. But our circle widens from day to day, and we have a duty to discharge to our new readers not less than to our friends of longer standing.

The evil of which we have written is so enormous and so nearly universal, that the reform involves a practical revolution. Of course this revolution cannot be wrought in a day or a month. In opposition to it we have the immense capital invested in the ordinary, hap-hazard system of making boots and shoes, the stolidity of the craft, and the utter ignorance of the world at large upon the science of anatomy and the wrongs induced by the old, barbaric method. But we have worked constantly and earnestly ever since the new truth dawned upon us, and from the day of our first publication on the subject, we have had great and growing proofs of the value of McComber's improvement and of our own power for good. Our influence has been exerted, not alone upon the wearers of shoes, but upon dealers as well. To interest dealers has been a constant and never-ceasing duty with us, and in this work we have been wonderfully successful. Hardly a day passes during which we do not receive the thanks of some maker or seller of boots and shoes for opening his eyes to the injury done by the old plan, and to the benefits resulting from the new. Added to all this, there comes the not unwilling confession that the adoption of the reform goods has largely increased business, even in a period of great financial depression. One dealer calls especially to thank us for our work, and to tell us that his conversion is radical and complete; that from being an entire unbeliever in the McComber plan, he has become convinced that the old system is a failure and a fraud; that from the small beginning of a half-dozen pairs, he has finally discarded all his old lasts, and now works only on McComber's; and that, from receipts averaging $50 a day, he now runs up to a daily business of nearly $800. Proofs such as these, of the growth of the new and better plan, are shown all about us, and bear testimony to the fact that our labors are pregnant with glorious results. When the pressure made by the public is sufficient to induce the dealer to order the McComber goods, it is pretty clear evidence that the potent leaven is actively at work; and it sustains the view which we took of the case las fall when we wrote that the time would surely come when the dealer in boots and shoes who adhered to the old deforming, murderous system would have to put up his shutters and take down his sign.

For, whatever may be the personal power and influence of any man, he cannot go on forever in the use of a false system, providing his neighbor has a better one. There is not power enough in all the world to induce the public to patronise a hatter whose hats must necessarily be "broken in" before they could be comfortably worn, provided another hatter could be found whose products were comfortable in the beginning. So it will be found in the case of the dealer in clothing for the feet; that which is comfortable, that which is right, will be purchased and favorably mentioned, while that which is distorting and radically wrong, will be avoided and condemned.

We are endeavoring to force the tradesmen in foot-clothing to adopt McComber's last and McComber's goods. We have no interest in McComber, except that we regard him as a reformer, and his work as among the most valuable of modern discoveries. He has systematized and perfected what before was vague and nonsensical. He has taken the old, ill-formed block of wood on which shoes were constructed, and has reduced it to scientific proportions, putting on here and taking off there; so that the shoe becomes a totally different structure—ready to receive the human foot and protect it from all external injury, without pressure and without distortion. He alone has recognized the fact that the foot has muscles which contract and expand with every motion; that these expansions and contractions occur many thousand times each day, in active life, and that provision must be made for this constant change, or the foot will suffer. As McComber has attained to perfection in this, so far as we are able to judge, we befriend him; but our interest in the reform itself is the interest of the public teacher who has a duty to perform which he would be cruel to neglect.

In view of our attitude upon this important topic, it will not be deemed surprising that we take special pleasure in the fact that a new acquisition has been made to the ranks of the converts to this religion of humanity and beauty—in the persons of a leading firm of Shoe Manufacturers, Messrs. Baldwin & Lamkin, Milford, Connecticut. There are several reasons for our satisfaction in this regard, one of which is that they were induced to adopt the system through the influence of our Journal, and the demand which had been created by our articles. Another reason—and one which greatly concerns the public at large—is, that this house manufactures boots and shoes of all kinds and sizes, from the creeping baby to the stalwart man—the only limitation being in the quality. Below a certain high standard of excellence they do not go; their goods are, therefore, denominated "first-class." But we are assured that they are the only manufacturers in America who produce everything needed by the public in first-class work. Those mothers who have written to us to know where little shoes, on McComber's plan, could be procured for creeping babies, will find their answer here. Those, too, who have asked us about slippers which would not spread over and wear through from contact of the slipper with the floor, will now be able to have this want supplied. Dealers who want a single pair to fit a customer whom they will call "notional," but who, in reality, is only sensible, will be glad to feel that by sending the measure to Baldwin & Lamkin, the single pair will be made and forwarded at the case price.

The goods made by this house arel all sewed either by hand or by machinery. The leather and material used is the best and only the best. They buy their Jodot Calf Skins and their French Kid, of Mr. L. Bailey the Sternfeldts, Mulford, Cary & Conkling; and their Sole-leather of J. B. Hoyt, all doing business in Spruce street, New York, and all of whom tell us that nothing but choice goods can be sold to Baldwin and Lamkin.

The motive for producing perfect work, resides with them in the fact that they have several large retail stores in the city of Boston and these must be supplied with the best articles which can be made. The direct and intimate relations which they thus bear to the consumer acquaints them with the public's demand, and they labor to meet that demaud in all possible ways. To do this has been a constant study for very many years. The partners are practical shoe-men, brought up to the business, and there is nothing connected with what Whittier terms "the gentle craft of leather," which they have not reflected upon and comprehended. For years they have sought to supply to their own customers as well as to the trade at large, boots and shoes for men and women, and for boys and girls of all ages, the durability of which, as well as the excellence of style and fit would commend them to the good judgement of customers. It is safe to say, that they have failed at only one point—the shape of the last. This failure they have had the intelligence to discover.

Mr. Baldwin has given us a sketch of his history, and has admitted the radical errors in the old last. He has told us that he had long given great attention to the subject of lasts, because satisfied that a grievous error existed somewhere. He experimented with new forms of lasts, constructed with his own hands, and was able to improve not a little on the old plan. But, as it subsequently appeared, all his improvements were in the direction of McComber's. On examining a McComber patent last, he discovered that he had been working with the same thought; lacking only the system which reduces McComber's plan to an exact science, so that one last or a million can be made under it with nothing to work by except the measurements. On discovering that he was simply feeling his way along a pathway previously fully explored, he sought out McComber and obtained the right to employ the perfect last in the extensive works of Baldwin & Lamkin at Milford.

We are impelled to commend this firm to public notice and confidence, not only on account of their high standing, as manufacturers and the excellent quality of the work which they turn out, but because of the great range of fine goods which their factory produces. Through them the needs of all can be supplied : and we hope the engrafting of the new method upon their solid groundwork of integrity and skill, will so rapidly heighten the dranghts upon them as to render a vast increase of space and machinery necessary to meet the enlarged demand.

